# Oxymetazoline Hydrochloride Eye-Drops as Treatment for Myasthenia Gravis-Related Ptosis: A Description of Two Cases

**DOI:** 10.7759/cureus.36351

**Published:** 2023-03-19

**Authors:** Mohamed Taha, Yuebing Li, John Morren

**Affiliations:** 1 Department of Neurology, Cleveland Clinic Neuromuscular Center, Cleveland, USA

**Keywords:** oxymetazoline hydrochloride, oxymetazoline, eyelid drooping, ocular myasthenia gravis, ptosis, myasthenia gravis

## Abstract

In this article, we described two patients with myasthenia gravis-related ptosis who experienced sustained improvement with the use of oxymetazoline hydrochloride ophthalmic solution 0.1%. Despite the commonly used treatments for ptosis in myasthenia gravis (MG), such as acetylcholinesterase inhibitors and corticosteroids, complete remission of ptosis is not always achieved, and these treatments are often accompanied by systemic side effects. Our case report suggests the long-term efficacy of daily use of oxymetazoline eye drops in improving ptosis, providing a potential alternative or adjunctive treatment option without significant adverse effects. Further research is necessary to confirm these observations across larger cohorts of MG patients and establish the effectiveness of oxymetazoline eye drops in MG-related ptosis.

## Introduction

Blepharoptosis (ptosis) is an abnormal downward displacement of the upper eyelid margin secondary to structural or neurological causes [1]. The primary muscles responsible for elevating the upper eyelid are a pair of skeletal muscles named levator palpebrae superioris, with a lesser contribution from the Müller's muscle, which is comprised of smooth muscle fibers innervated by the sympathetic nervous system through adrenergic receptors. Blepharoptosis can be classified into two categories: congenital and acquired. Acquired ptosis encompasses a range of underlying causes, including neurogenic, traumatic, aponeurotic, and myogenic causes, with the latter being a potential result of myasthenia gravis (MG) [1].

Myasthenia gravis is a chronic autoimmune disorder characterized by fatigable muscle weakness secondary to neuromuscular junction dysfunction. The most commonly observed symptoms of MG are ocular in nature, with ptosis and diplopia being the initial presentation in over half and eventually present in over 90% of MG patients [2,3]. Ptosis can have a significant impact on the patient's vision, leading to difficulties in performing daily activities such as driving or reading, as well as having a negative impact on the patient's well-being. Ptosis has been shown to cause increased anxiety and social avoidance [4].

Despite the prevalence of ptosis in MG, there is a relative paucity of randomized trials on the management of ocular MG. The available treatment options for ptosis associated with MG include pyridostigmine, prednisone, and surgical eyelid correction. In a randomized, double-blind study, it was found that 83% of patients with ocular MG showed a positive response to prednisone [5]. Another study found that 29% of patients experienced resolution of their ocular symptoms with the use of pyridostigmine, while 70% of patients who also used prednisone showed improvement [6]. A separate study reported the resolution of unilateral ptosis in 85% of patients treated with prednisone alone and 50% of patients treated with pyridostigmine alone [7]. However, the use of pyridostigmine and prednisone is associated with potentially unpleasant and sometimes serious side effects. Thymectomy has been shown to be effective in treating generalized MG [8] but is generally not recommended for ocular MG. The role of newly developed complement inhibitor and neonatal Fc receptor for IgG (FcRn) inhibitor therapies in the treatment of ocular MG is uncertain as these treatments have not been studied in this context, and the cost of these therapies is likely to be prohibitively high. Therefore, more cost-effective and reasonably safe treatment options are needed for managing ocular MG or generalized MG with prominent ocular features.

Oxymetazoline hydrochloride

Oxymetazoline hydrochloride is a non-selective alpha-adrenergic agonist that stimulates both alpha-1 and alpha-2 receptors [9], including those found in the Müller's muscle of the eyelid. The utilization of oxymetazoline hydrochloride ophthalmic solution 0.1% for the treatment of acquired blepharoptosis (ptosis) received approval from the U.S. Food and Drug Administration (FDA) in July 2020. The standard dosing is one drop of the solution in the affected eye one time each day. Only one case of a 68-year-old MG patient with temporary improvement of ptosis for seven hours after using topical ocular oxymetazoline has been documented to date [10]. However, the long-term efficacy of the drug for ptosis or its impact on MG treatment has not yet been described. In this study, we describe two patients who experienced long-term improvement of ptosis with the regular use of oxymetazoline.

## Case presentation

Case one

**Figure 1:**
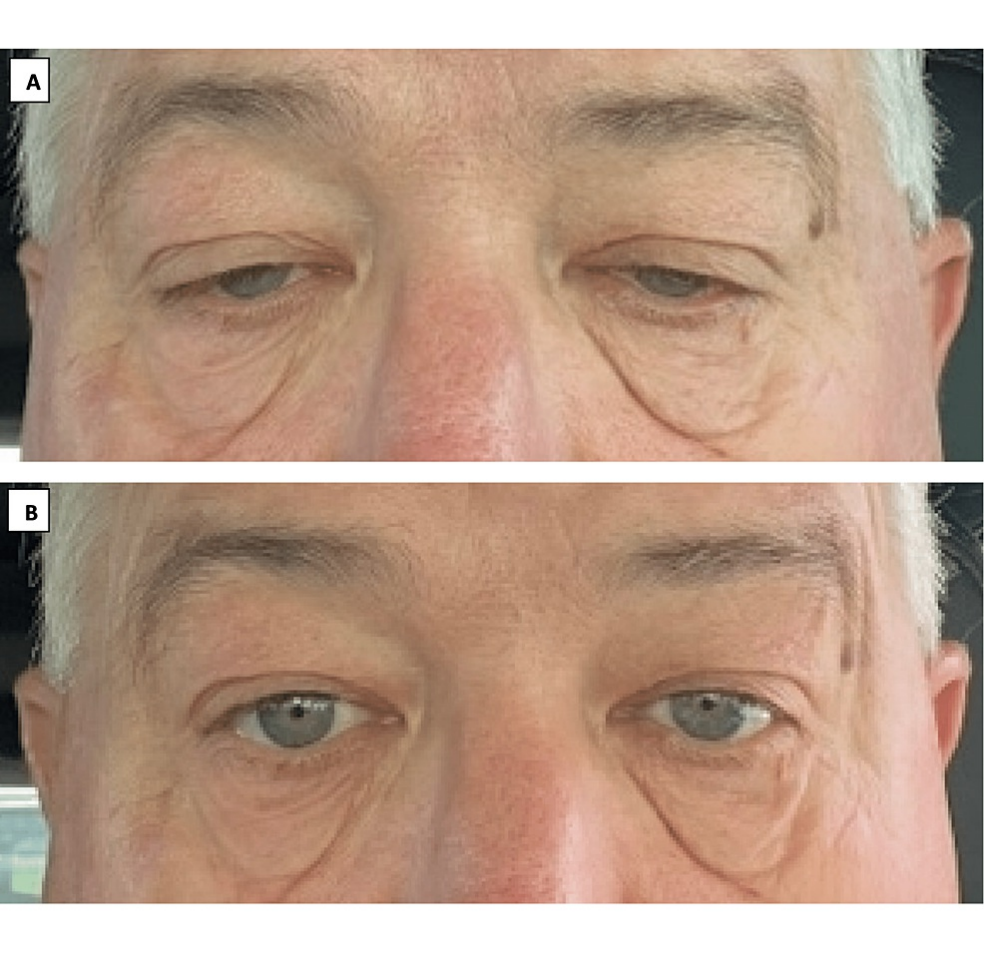
Pictures demonstrate the presence of baseline bilateral eyelid ptosis in image A, and the improvement of ptosis 30 mins after instillation of oxymetazoline hydrochloride 0.1% ophthalmic solution in image B. Used with patient permission.

A 66-year-old man presented for evaluation of ocular and mild dysarthria and dysphagia with fatigable weakness. MG was confirmed with the presence of positive acetylcholine receptor binding antibodies, as well as abnormal decrement on repetitive nerve stimulation electrodiagnostic testing. The patient’s symptoms included drooping of both eyelids, with the left worse than the right and worsening as the day went on. His ptosis did not improve with treatment of pyridostigmine (120 mg every four to five hours) and prednisone (10mg-30 mg daily) over several months. However, oxymetazoline hydrochloride 0.1% ophthalmic solution was helpful in alleviating ptosis in addition to the above treatment. Improvement of ptosis occurred within 30 minutes of each application to the eye and lasted for an average of six to eight hours (Figure 1). On average, a dosage of once daily was sufficient, and the patient found it particularly helpful when used prior to vision-intensive activities such as driving. The patient has been using oxymetazoline eye drop for two months without noticing a waning efficacy or significant adverse effects.

Case two

A 44-year-old woman presented with a three-year history of ptosis and double vision. The eyelid drooping was worse on the left side and worsened throughout the day. The patient also reported constant double vision, most noticeable while driving. No difficulties with swallowing, breathing, or limb movement were noted. Serum testing for antibodies showed that acetylcholine receptor, muscle-specific kinase (MuSK), and low-density lipoprotein receptor-related protein 4 (LRP4) were all negative. Single fiber electromyography findings were consistent with a neuromuscular junction transmission disorder, supporting the diagnosis of seronegative MG. The patient was started on pyridostigmine (60mg) three times per day and prednisone (20mg) daily.

Despite taking pyridostigmine and prednisone for several months, the patient reported no improvement in ocular symptoms, particularly ptosis, and minimal improvement in non-ocular symptoms, such as generalized weakness. However, she reported improvement in ptosis with the use of oxymetazoline hydrochloride 0.1% ophthalmic solution. Daily morning use of the oxymetazoline eye drops led to a prolonged sustained improvement in ptosis for 14 months. The patient was able to reduce and eventually discontinue usage of prednisone and pyridostigmine due to side effects, including weight gain and irritability. Although there was no impact on diplopia, with the marked improvement of ptosis using oxymetazoline eye drops, the patient was able to participate in daily activities that were previously difficult, such as driving and working a full-time job.

## Discussion

Some patients with ocular manifestations of MG do not favorably respond to standard treatments, despite high doses of immunosuppressive medications and/or pyridostigmine and may experience adverse effects as a result. Here we describe two patients with ocular symptoms from MG who experienced an improvement in ptosis after using oxymetazoline hydrochloride 0.1% ophthalmic solution.

Oxymetazoline has a rapid onset of action (5-10 minutes), and its duration of action is estimated to last between five and six hours [11]. It has been utilized in a range of therapeutic applications, such as the treatment of nasal decongestion [11], ocular presbyopia [12], mild ocular redness [13], and ptosis [14]. The results of a meta-analysis of two randomized, double-blinded clinical trials demonstrated that the use of oxymetazoline eye drops is both effective and safe in the treatment of acquired ptosis, mostly due to aging [14]. A total of 304 participants were enrolled and treated with oxymetazoline once daily for 42 days. The modified visual field test for ptosis, as measured by the Leicester Peripheral Field Test, demonstrated statistically significant improvement with oxymetazoline compared to placebo, as shown by the following results: on day one, the mean difference was 4.07 (95%CI, 2.74-5.39), with a mean score of 5.9 (SD = 6.4) for oxymetazoline and 1.8 (SD =4.1) for placebo (p<.001). On day 14, the mean difference was 4.74 (95%CI, 3.43-6.04), with a score of 7.1 (5.9) for oxymetazoline and 2.4 (5.5) for placebo (p<.001). The study reported minimal side effects, including mild conjunctival hyperemia or punctate keratitis [14]. The incidence of adverse events in the oxymetazoline group was found to be lower than in the placebo group (31% vs. 35.6%), with the majority of side effects reported being mild in intensity (81%). However, patients with ptosis due to MG were excluded from both trials. Our case report is significant in describing the sustained efficacy of oxymetazoline in patients with ptosis related to MG. We have demonstrated that the use of oxymetazoline eye drops is helpful in relieving MG-related ptosis even when more standard MG treatments are not successful, and its efficacy is quick in onset and long-lasting without significant adverse effects. 

**Figure 2:**
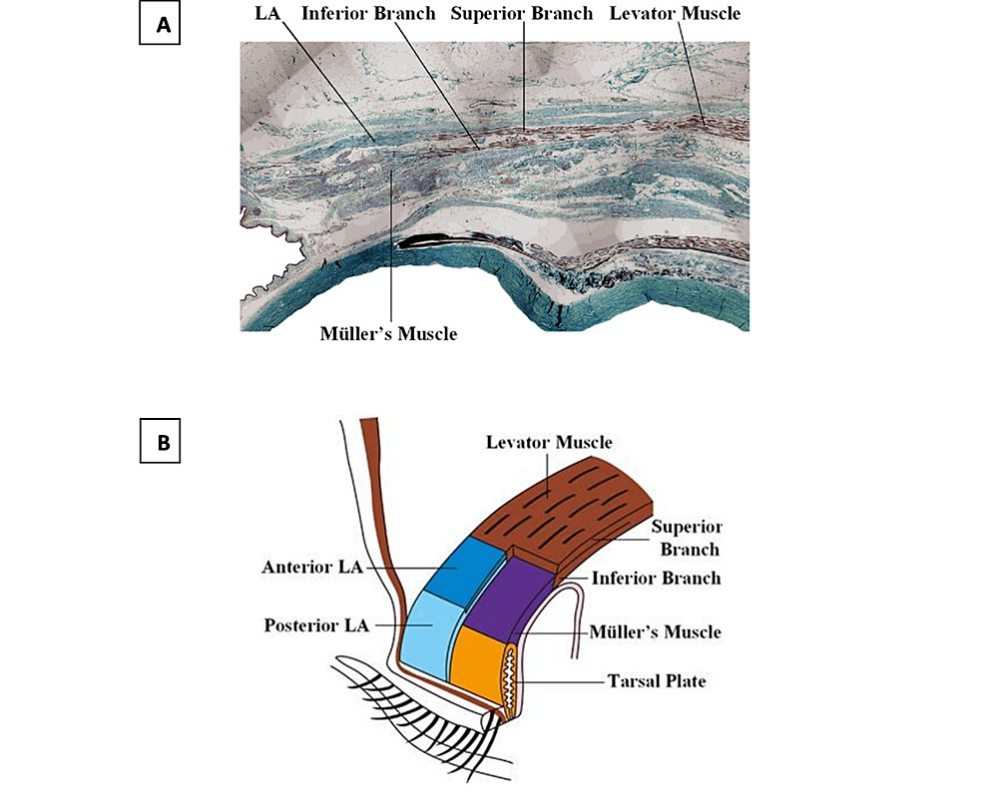
Anatomical picture (sagittal section) (A) and diagram (B) showing the striated LPSM divided into two branches in the distal area The Müller smooth muscle originates from the inferior branch of the LPSM and connects the LPSM to the superior tarsal plate in mediating upper eyelid elevation. In portion B, part of the distal superior branch of the LPSM is removed for better viewing. LA - levator aponeurosis, LPSM - levator palpebrae superioris muscle Modified from Kakizaki et al. [18], used with permission.

The exact mechanisms by which oxymetazoline works to improve ptosis in MG patients remain unclear. There are two possible explanations for its effectiveness. Firstly, oxymetazoline may act on the Müller's muscle by activating its alpha-adrenergic receptors, causing contraction of the smooth muscle and correction of ptosis. This putative mechanism would also explain how similar ophthalmic alpha-adrenergic agonist compounds produce improvement in cases of iatrogenic ptosis from botulinum toxin injections [15,16]. Since eyelid-elevating smooth muscle is the target of alpha-adrenergic agonists, the issue of impairment in neuromuscular junction transmission (relevant only to skeletal muscle) is circumvented. Secondly, Müller's muscles may act as muscle spindles and mediate proprioception sense to the fast-twitch and slow-twitch fibers in the levator palpebrae superioris muscles, as these two muscles are physically connected (Figure 2) [17]. Stimulation of the Müller's muscle may result in stimulation of the slow-twitch fibers in the levator palpebrae superioris muscle, leading to improvement of the ptosis [17].

The quick effect onset of oxymetazoline, its sustained efficacy, ease of administration, and lack of predominant systemic side effects make it a promising treatment option for ptosis caused by MG, especially in patients who are intolerant of or reluctant to use conventional MG medications. However, oxymetazoline is not known to have any impact on diplopia. Nonetheless, as seen in case two, improvement of marked ptosis can be sufficient to restore meaningful engagement in activities of daily living, even if diplopia persists. More extensive research through larger series or randomized controlled trials is necessary to fully determine its usefulness, particularly in the MG patient population.

## Conclusions

In conclusion, our case report highlights the potential of oxymetazoline hydrochloride ophthalmic solution 0.1% as a safe and effective treatment option for myasthenia gravis-related ptosis. Despite commonly used MG treatments, complete remission of ptosis is not always achievable, and the associated side effects can be considerable. Our two MG patients showed sustained improvement of ptosis with daily use of oxymetazoline eye drops, suggesting a promising alternative or adjunctive treatment option. Further research with larger cohorts of MG patients is needed to confirm these findings and more definitively establish the effectiveness of oxymetazoline eye drops in managing MG-related ptosis.
